# DNA methylation of the *KLK8* gene in depression symptomatology

**DOI:** 10.1186/s13148-021-01184-5

**Published:** 2021-10-29

**Authors:** Anna Starnawska, Lina Bukowski, Ana Chernomorchenko, Betina Elfving, Heidi Kaastrup Müller, Edwin van den Oord, Karolina Aberg, Jerry Guintivano, Jakob Grove, Ole Mors, Anders D. Børglum, Anders L. Nielsen, Per Qvist, Nicklas Heine Staunstrup

**Affiliations:** 1 Department of Biomedicine, Aarhus University, Hoegh Guldbergs Gade 10, 8000 Aarhus C, Denmark; 2 The Lundbeck Foundation Initiative for Integrative Psychiatric Research, iPSYCH, Aarhus, Denmark; 3 Center for Genomics and Personalized Medicine, CGPM, Center for Integrative Sequencing, iSEQ, Aarhus, Denmark; 4 Translational Neuropsychiatry Unit, Aarhus University, Aarhus, Denmark; 5 Center for Biomarker Research and Precision Medicine (BPM), School of Pharmacy, Virginia Commonwealth University, Richmond, USA; 6 Department of Psychiatry, University of North Carolina at Chapel Hill, Chapel Hill, USA; 7 Bioinformatics Research Centre, Aarhus University, Aarhus, Denmark; 8 Psychosis Research Unit, Aarhus University Hospital, Aarhus, Denmark

**Keywords:** Epigenetics, DNA methylation, KLK8, Neuropsin, Depression

## Abstract

**Background:**

Depression is a common, complex, and debilitating mental disorder estimated to be under-diagnosed and insufficiently treated in society. Liability to depression is influenced by both genetic and environmental risk factors, which are both capable of impacting DNA methylation (DNAm). Accordingly, numerous studies have researched for DNAm signatures of this disorder. Recently, an epigenome-wide association study of monozygotic twins identified an association between DNAm status in the *KLK8* (neuropsin) promoter region and severity of depression symptomatology.

**Methods:**

In this study, we aimed to investigate: (i) if blood DNAm levels, quantified by pyrosequencing, at two CpG sites in the *KLK8* promoter are associated with depression symptomatology and depression diagnosis in an independent clinical cohort and (ii) if *KLK8* DNAm levels are associated with depression, postpartum depression, and depression symptomatology in four independent methylomic cohorts, with blood and brain DNAm quantified by either MBD-seq or 450 k methylation array.

**Results:**

DNAm levels in *KLK8* were not significantly different between depression cases and controls, and were not significantly associated with any of the depression symptomatology scores after correction for multiple testing (minimum *p* value for *KLK8* CpG1 = 0.12 for ‘Depressed mood,’ and for CpG2 = 0.03 for ‘Loss of self-confidence with other people’). However, investigation of the link between *KLK8* promoter DNAm levels and depression-related phenotypes collected from four methylomic cohorts identified significant association (*p* value < 0.05) between severity of depression symptomatology and blood DNAm levels at seven CpG sites.

**Conclusions:**

Our findings suggest that variance in blood DNAm levels in *KLK8* promoter region is associated with severity of depression symptoms, but not depression diagnosis.

**Supplementary Information:**

The online version contains supplementary material available at 10.1186/s13148-021-01184-5.

## Introduction

Depression is a common, multifactorial, and devastating mental disorder. It is among the leading causes of disability worldwide, with a lifetime prevalence estimated at ~ 14%, and even reaching 21% in high-income countries [1, 2]. Liability to depression in part constitutes the extreme of a quantitatively measurable depression symptomatology, where severity varies across the population [3–6]. The risk of depression and severity of depression symptomatology are both heritable traits. According to twin studies, 40% of the variation in depression liability is attributed to additive genetic effects [7–9]. The genetic risk of developing depression is, similarly to other common multifactorial diseases, based on common genetic variation of small effects at multiple loci across the genome. Accordingly, a recent meta-analysis study identified 102 genome-wide significant depression-susceptibility loci [10]. Apart from the genetic variation, environmental exposures, such as social, socioeconomic, and lifestyle factors, play important roles in the etiology of the disorder [11–14].

Due to the complex etiology of depression, many studies have sought to identify its peripheral biomarkers to allow for improved and earlier diagnosis, as well as monitoring of the disorder. This includes a rapidly growing number of studies on DNA methylation (DNAm), since both genetic and environmental risk factors modulate its variance across the genome [15, 16]. A recent epigenome-wide association study (EWAS) performed in blood of 724 Danish monozygotic twins reported DNAm levels in the promoter region of Kallikrein Related Peptidase 8 (*KLK8*, neuropsin) to be associated with severity of depression symptomatology in the general population [17]. Along this line, increased expression levels of *KLK8* in blood were shown to be significantly associated with recurrent depression diagnosis in comparison with patients with first episode of depression [18], and to healthy controls [19]. The 19q13 genomic region, in which *KLK8* is located, has been implicated in both schizophrenia and bipolar disorder by genetic linkage studies [20, 21], and genetic common variation in *KLK8* has been associated with bipolar disorder and cognitive functioning [22]. Moreover, environmental risk factors for depression, like acute stress, lead to increased *KLK8* expression levels in hippocampus [23], where it plays a role in proteolysis of trophic factors and synaptic plasticity [24]. The neurobiology of *KLK8* is thus functionally related to that of established biomarkers of depression, like Brain-Derived Neurotrophic Factor (BDNF) and Vascular Endothelial Growth Factor (VEGF) [25]. Notably, inactivation of *KLK8* has protective effects against depressive-like behaviors and memory impairment initially induced by chronic stress in mice [23, 26]. A more comprehensive review of the role of *KLK8* in mental health phenotypes is available elsewhere [27].

In this study, we aimed at testing if DNAm levels in the promotor region of *KLK8* have clinical applicability in stratifying depression cases based on their depression severity and to distinguish cases from unaffected controls. The study was performed in a subsample (*n* = 160) of individuals from an independent Danish clinical Psychosocial RIsk factors for Stress and MEntal disease (PRISME) cohort with a custom-made pyrosequencing assay targeting the promoter region of *KLK8*. Additionally, we tested the association between *KLK8* DNAm levels in four independent methylomic cohorts with data on depression and depression-related phenotypes.

## Materials and methods

### Cohort description

Individuals included in this study represent a subsample of the Danish PRISME cohort created to investigate impact of work-related psychological strain on the risk of developing stress, burn-out, and depression comprising 4448 individuals [28, 29]. Our study was performed on 80 depression cases and 80 gender-matched controls drawn from the PRISME cohort. Depression in PRISME was diagnosed through diagnostic SCAN interview (Schedules for Clinical Assessment in Neuropsychiatry) according to the ICD-10 criteria, as described before [28, 29]. Genomic DNA extracted from peripheral blood was available from previous studies on the PRISME cohort [30–32].

### DNAm assay

Genomic DNA for all study participants was bisulfite converted (Qiagen, Hilden, Germany) according to manufacturer’s protocol. DNAm levels at CpG site chr19: 51,504,987 and the EWAS-candidate CpG site for depression symptomatology (cg05777061) [17], both located in the promoter region of *KLK8*, were quantified with a custom-made pyrosequencing assay. We refer to these two sites as CpG1 and CpG2 (cg05777061), respectively. To amplify the bisulfite-converted sequence containing CpG1 and CpG2 sites (5′ GGG CGG AGG GGA TTG AAC GT 3′ with CG-sites underlined), a forward PCR primer (5′ GAT TTT GGA GTT TTT TAA TTG GGA A 3′) and a biotinylated reverse PCR primer (5′ [BIO] ATC CCT CCT CTC CCT AAC CTC 3′) were designed (Additional file 1: Fig. S1). PCR amplification of the region of interest was performed according to conditions described in Additional file 2: Table S1. The biotinylated PCR product was sequenced using a sequencing primer 5′ GTG AGT GAG AAG 3′ with Pyromark Q24 sequencer (Qiagen, Hilden, Germany). This single pyrosequencing assay provided information on DNAm levels at both CpG1 and CpG2, both located in the *KLK8* promoter region and separated by 14 bp, as depicted by Additional file 1: Fig. S1 and Additional file 3: Fig. S2. All 160 PRISME samples were pyrosequenced in duplicates according to manufacturer’s protocol (Qiagen, Hilden, Germany). Samples of depression cases and controls were intermixed throughout plates and were processed in two batches. Data obtained from the pyrosequencing experiment were further filtered based on quality control information provided by the pyrosequencer on performance of each sample. The quality control assessment, generated by the PyroMark software (Qiagen, Hilden, Germany), is undertaken by setting the non-CpG dispensations as reference peaks and measuring how well they conform to the theoretical pyrogram generated from the original sequence [33]. Samples marked in red in the pyrosequencer quality report (corresponding to low quality data) were removed from the dataset. For the remaining samples, dataset was split into CpG1 and CpG2 and was filtered to keep only samples with both replicates available and DNAm replicate difference < 5%. For the remaining good quality data, DNAm mean was calculated between duplicates and this value was used for all statistical analyses. Overview of quality control steps and number of remaining data points for CpG1 and CpG2 is provided by Additional file 4: Fig. S3.

### Statistical analyses in PRISME

The statistical analyses for PRISME cohort in this study were performed in three steps: (i) confirmation of clinical differences between depression cases and controls, (ii) testing for associations between DNAm levels at CpG1 and CpG2 and depression symptomatology severity for 20 SCAN variables from section 6 ‘Depressed mood and ideation’ in cases only, (iii) testing DNAm differences at CpG1 and CpG2 between depression cases and controls.

(i) We first evaluated if depression symptomatology scores (SCAN items v6_001: Depressed mood, v6_003: Tearfulness and crying, v6_004: Anhedonia) were significantly different between the 80 depression cases and 80 controls selected from the PRISME cohort. These were the only three SCAN variables where information on depression symptomatology scores was available for all study participants, except for one control individual. Three analyses were performed, one for each variable (v6_001, v6_003, and v6_004), and each test was performed with the use of generalized linear model. Continuous depression symptomatology scores were used as the predictor variable, case–control status as the outcome variable and the model was adjusted for sex according to the following formula:
  $${\text{case - control status}}\sim {\text{depression symptomatology variable}} + {\text{sex}}$$
(ii) We next investigated the link between DNAm levels of *KLK8* and severity of depression symptoms within PRISME depression cases. Multicollinearity was calculated between the 20 SCAN section 6 (depressed mood and ideation) variables to evaluate how many independent depression scores are available for this analysis. Number of independent tests was estimated based on the correlation matrix with the use of Nyholt method by using *meff()* function implemented in poolR package [34]. By linear regression, we tested the association between each of the 20 depression symptomatology variables and DNAm levels at CpG1 and CpG2 according to the following formula:
  $${\text{depression symptomatology variable}}\sim {\text{DNAm}} + {\text{sex}} + {\text{age}} + {\text{processing batch}}$$
  Since sex, age, and processing batch are known to impact DNAm levels, they were included in the model to reduce their possible confounding effects in this study [35, 36].
(iii) We next extended the analysis to include both PRISME cases and controls, where, with the use of generalized linear model, we tested the association between DNAm levels at CpG1, CpG2, and depression case–control status, while adjusting for sex, age, and processing batch according to the following formula:
  $${\text{case - control status}}\sim {\text{DNAm}} + {\text{sex}} + {\text{age}} + {\text{processing batch}}$$

All statistical analyses in this study were performed in R statistical environment [37].

### Statistical analyses in NESDA, BioMom, GSMS, and BrainMDD cohorts

In order to evaluate the association between DNAm of *KLK8* in blood and depression, we also analyzed data from four other independent methylomic datasets:

1. NESDA, The Netherlands Study of Depression and Anxiety, involved major depressive disorder (MDD) cases and controls (*n* = 1132) [38–40]. Participants were aged 18–65 years at baseline and were followed for 8 years with assessments every 2 years. MDD was diagnosed using the DSM-IV-based Composite International Diagnostic Interview (version 2.1) [41]. DNAm data were generated using blood samples collected at baseline.
2. BioMom study (*n* = 1400) involved postpartum depression (PPD) cases and controls aged 17–44 years [42]. PPD was determined 6 weeks postpartum using the structured diagnostic psychiatric interview MINI-plus, and DNA was obtained from participants’ blood samples [43].
3. GSMS (*n* = 560 individuals at multiple time points resulting in a total of 1034 assays) involved participants aged 9–13 at intake and followed for > 25 years, with a median number of 8 assessments per participant [44, 45]. Depression symptoms were assed with the CAPA/YAPA [46, 47], and DNAm levels were assayed from blood.
4. BrainMDD (*n* = 206) cohort originated from meta-analysis initiative of 206 postmortem brain samples from 3 brain collections, as previously described [48, 49]. The sample collections were predominantly from Australia (30 MDD, 31 control; Brodmann area (BA) 25), USA (44 MDD, 37 control; BA10), and Canada (39 MDD, 25 control; BA10).

All four cohorts represent studies where association was tested between depression-related phenotypes and DNAm levels quantified in blood (NESDA, BioMom, GSMS) or brain (BrainMDD) samples. Methylomic data for these cohorts were generated by methyl-CG binding domain sequencing (MBD-seq) method, with exception for the BioMom cohort which used methylomic array [42].

For GSMS and NESDA, the methylome was assayed with an optimized protocol [50, 51] for MBD-seq [52]. Bowtie2 [53] was used for local and gapped alignment of reads. Quality control was performed for reads, samples, and methylation sites using methods described elsewhere [54]. For BioMom, data were generated using the Illumina Infinium Human Methylation 450K BeadChip and quality control of the data was performed as discussed previously [55]. Association tests for all three studies were performed using multiple regression analyses in RaMWAS [52, 56] with four sets of covariates. First, we regressed out assay-related variables (i.e., potential technical artifacts) such as sample batches and enrichment efficiency [56]. Second, we included age, age squared, sex and race as covariates. Third, to avoid false positives due to cell-type heterogeneity, we regressed out cell-type proportions of the four common cell types in blood that were estimated using a reference panel [57]. Fourth, we performed a principle component analysis after regressing out all the measured covariates from the methylation data. Based on a scree test [58], the first principal components were used to account for remaining unmeasured sources of variation. Power calculations for each tested cohort (PRISME, NESDA, GSMS, BioMom, and BrainMDD) used in this study to identify the association between *KLK8* DNAm levels and depression-related phenotypes are provided in Additional file 2: Table S3.

## Results

### Sample demographics

Overview of demographics for all PRISME individuals included in this study, together with their differences on the three SCAN variables between depression cases and controls (v6_001, v6_003, v6_004), is available in Table 1 (Materials and Methods: i). We confirmed that depression cases had significantly higher depression symptomatology scores among all three tested SCAN variables in comparison with controls in the PRISME sub-cohort assessed in this study (Table 1). Among included individuals, the majority were woman (80%), but there was no statistical difference in frequency of males or age distribution between depression cases and controls (Table 1, *p* value > 0.05).

**Table 1:** Overview of PRISME sample demographics

	Cases	Controls	*P* value
Sex (females/males)	67/13	62/18	0.4^a^
Mean age in years (SD)	45.4 (9.1)	44.8 (10.5)	0.7^b^
SCAN v1_001: depressed mood, mean (SD)	2.0 (0.7)	0.3 (0.6)	1.60E−37^b^
SCAN v6_003: tearfulness and crying, mean (SD)	1.4 (0.9)	0.1 (0.4)	8.70E−22^b^
SCAN v6_004: anhedonia, mean (SD)	1.6 (0.9)	0.1 (0.3)	6.24E−30^b^
Methylation CpG1, mean (SD)	62.6 (6.1)	60.1 (9.0)	2.50E−01^b^
Methylation CpG2, mean (SD)	17.2 (4.1)	16.6 (3.7)	4.90E−01^b^

^a^Chi-square test

^b^Generalized linear regression

After quality control, DNAm pyrosequencing data were available for 87 study participants for CpG1 (43 cases, 44 controls), and 133 for CpG2 (71 cases, 62 controls) (Additional file 4: Fig. S3). Filtering out the methylation data due to quality control did not induce any strong sex or age biases in neither CpG1 nor CpG2 datasets (*p* values > 0.05, Additional file 2: Table S2). DNAm levels at CpG1 and CpG2 were significantly correlated (Pearson’s correlation = 0.7, *p* value = 4.02E − 14).

### *KLK8* DNAm in depression symptomatology and diagnosis

Since the initial association between *KLK8* DNAm levels and depression phenotypes was discovered not for the depression ICD-10 diagnosis but for severity of depression symptoms, we first evaluated association between DNAm levels at CpG1 and CpG2 with the 20 SCAN variables for depression symptomatology (Materials and Methods: ii). Description of each tested SCAN variable, together with overview of completeness of this clinical data, is provided in Table 2.

**Table 2:** Overview of SCAN variables from section 6 ‘Depressed mood and ideation’ for 160 studied PRISME individuals

Variable	Variable description	Completeness rate (case/control)
v6_001	Depressed mood	1/0.99
v6_003	Tearfulness and crying	1/0.99
v6_004	Anhedonia	1/0.99
v6_005	Duration of depressed mood or anhedonia	0.98/0.44
v6_006	Loss of hope for the future	0.99/0.13
v6_007	Feeling of loss of feeling	0.99/0.11
v6_008	Loss of reactivity	0.99/0.1
v6_009	Morning depression	0.99/0.09
v6_010	Preoccupation with death or catastrophe	0.99/0.09
v6_011	Suicide or self-harm	0.99/0.09
v6_012	Tedium vitae	0.99/0.09
v6_013	Pathological guilt	0.99/0.11
v6_014	Guilty ideas of reference	0.99/0.1
v6_015	Loss of self-confidence with other people	0.99/0.11
v6_016	Social withdrawal	0.99/0.09
v6_017	Loss of self-esteem	0.99/0.1
v6_018	Delusions of guilt or worthlessness in context of depression	0.73/0.04
v6_019*	Delusions of catastrophe in context of depression	0.69/0.04
v6_020	Hypochondriac delusions in context of depression	0.85/0.04
v6_025	Age of first onset of depression symptoms	0.86/0.09
v6_026b*	Date of onset of psychotic symptoms or psychotic episode	0.93/0.3
v6_027	Interference of activities due to depression	0.95/0.26

^*^Variables omitted from the analysis due to 0 variance in the measurements

**Fig. 1:**
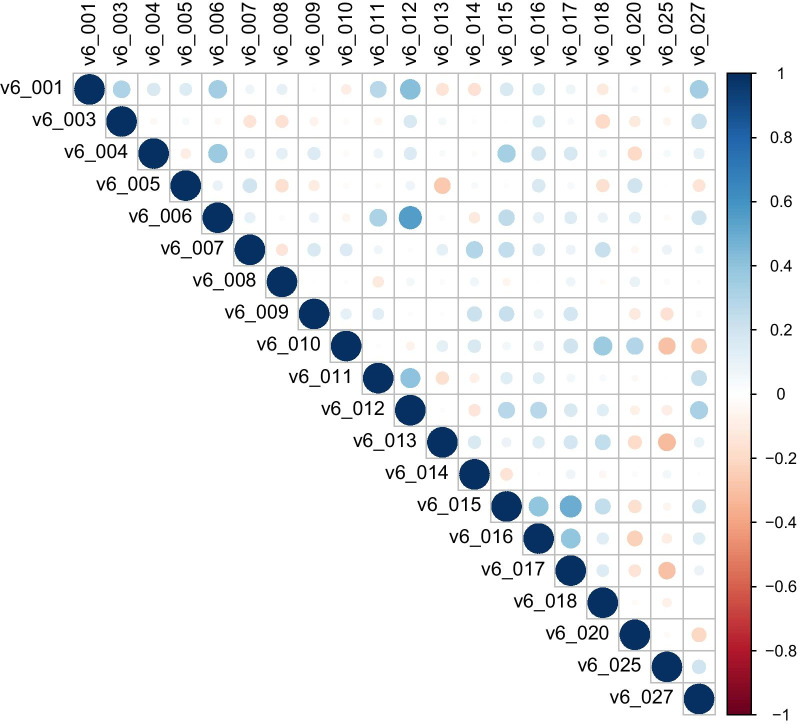
Multicollinearity of 20 SCAN depression scores among PRISME cases (*n* = 80)

Although 22 SCAN v6 traits were measured for PRISME individuals, only 20 of them had completeness rate > 75% for cases and standard deviation > 0 (Table 2). Overview of correlation structure between the included 20 SCAN covariates in PRISME cases is visualized in Fig. 1. The plot indicates that even though there is some level of correlation between the 20 SCAN depression variables included in the study, the correlation between the majority of the pairwise comparisons was rather low. The highest positive correlation (0.55) was observed between v6_006 (loss of hope for the future) and v6_012 (tedium vitae), which are two conceptually related questions (Fig. 1). This was followed by correlation between v6_015 (loss of self-confidence with other people) and v6_017 (loss of self-esteem) (0.5), and correlation between v6_001 (depressed mood) and v6_012 (tedium vitae) (0.43). The strongest negative correlation was observed between v6_025 (age of onset of depressive symptoms) and v6_013 (pathological guilt) (− 0.31), closely followed by its (v6_25) correlation with v6_017 (loss of self-esteem) (− 0.3) and v6_10 (preoccupation with death or catastrophe) (− 0.29) (Fig. 1). Analysis of number of effective tests identified 19 independent variables across the 20 SCAN depression symptomatology scores.

We next tested the association between each of the 20 SCAN variables and DNAm levels at CpG1 and CpG2 among depression cases (Materials and Methods: ii). The lowest p value for CpG1 (0.12) was observed for the v6_001 (depressed mood) with higher methylation observed for the more severe score for this trait. For CpG2, the lowest *p* value (0.03) was observed for v6_015 (loss of self-confidence with other people), with lower methylation observed for the more severe score. This was the only finding with *p* value < 0.05 from the association analysis between 20 SCAN depression symptomatology scores and DNAm at CpG1 and CpG2, but it did not remain significant after correction for multiple testing (significance threshold *p* value = 0.0026 for an estimated 19 independent tests).

Next, we tested if DNAm levels at CpG1 or CpG2 are associated with the depression case–control status (Materials and Methods: iii). Even though mean methylation at CpG1 and CpG2 was higher in depression cases in comparison with controls (regression estimate_CpG1_ = 1.5 and estimate_CpG2_ = 0.45), we did not find a statistically significant difference between methylation levels at these two sites to be associated with ICD-10 depression diagnosis in the PRISME sample (Table 1).

### Association analyses between *KLK8* DNAm and depression-phenotypes

**Fig. 2:**
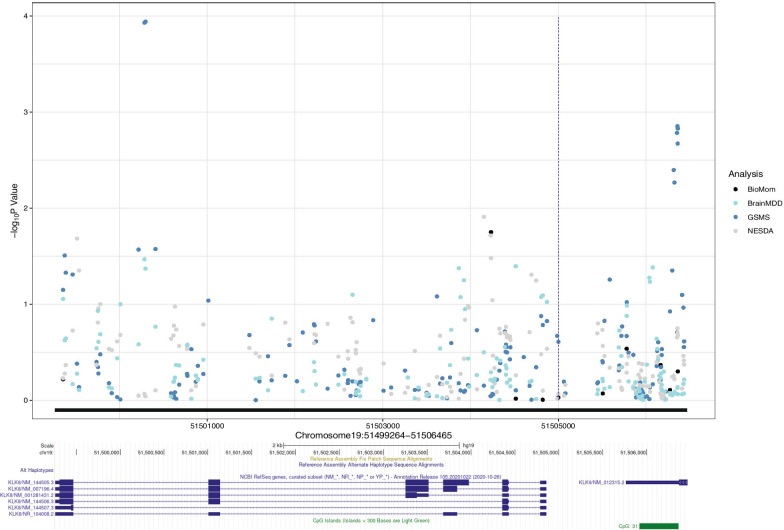
Regional association plot depicting results for the association between DNAm levels in *KLK8* and its promoter region (from 1500 bp upstream the transcription start site) and depression-related phenotypes in the NESDA, BioMom, GSMS, and BrainMDD cohorts. Black line along x-axis marks the investigated region (chr19:51,499,264–51,506,465, hg19) in the four methylomic cohorts, the genomic region spans the *KLK8* gene and its promoter. Gene tracks with *KLK8* transcripts for this region are presented according to RefSeq Curated (hg19). Blue dotted line marks position of the cg05777061 probe

We further tested the association between *KLK8* DNAm levels and depression-related phenotypes in four methylomic cohorts, with data on CpG1 site available in NESDA and GSMS, and on CpG2 site in NESDA, GSMS, and BioMom. Figure 2 provides an overview of all results generated by analyses in all four cohorts for the *KLK8* gene body and promoter region (+ 1500 bp from transcription start site). The most significant signals in the *KLK8* gene body and in the promoter region were observed in the GSMS cohort, where blood DNAm levels were tested for association with depression symptomatology. The most significant findings in *KLK8* gene body were observed for CpG sites chr19: 51,500,297 (*p* value = 0.00011) and chr19: 51,500,285 (*p* value = 0.00012), and in promoter region at chr19: 51,506,358 (*p* value = 0.0014) and chr19: 51,506,362 (*p* value = 0.0015). The association between depression severity score and variation of DNAm levels in the *KLK8* promoter region was supported by 7 CpG sites with *p* value < 0.05 in the GSMS cohort (Fig. 2).

## Discussion

In recent years, the number of EWASs in the field of mental health has increased significantly and identified new candidate genes associated with mental disorder symptoms, diagnosis, and their trajectory [17, 59–62]. However, in contrast to findings from epigenetic studies of other complex diseases (e.g., cancer [63]), their biological relevance and clinical applicability are rarely, if ever, properly assessed. In this study, we aimed at testing if epigenetic variation in the *KLK8* promoter region, recently implicated with depression symptomatology through EWAS [17], is associated with depression severity scores and depression case–control status first in an independent clinical PRISME cohort. The test was performed for two CpG sites, the candidate locus from depression symptomatology EWAS (CpG2) and its neighboring site (CpG1) located in *KLK8* promoter region. In the PRISME cohort, we observed a tendency of higher DNAm levels at CpG1 in *KLK8* promoter region to be associated with more severe depression symptoms. The same direction of effect was observed for seven CpG sites in the *KLK8* promoter region in the much larger GSMS cohort, where higher DNAm levels were significantly associated with more severe depression symptomatology. However, even though our study in PRISME and GSMS cohorts suggest that DNAm variation in the *KLK8* promoter region may contribute to severity of depressed mood, the direction of association observed in these studies is opposite to the one reported previously in the monozygotic twin study [17]. It is also important to note that the CpG site identified to be significant in the EWAS (cg05777061) was not significantly associated with depression symptomatology in neither PRISME nor GSMS.

There are several crucial differences between characteristics of the cohorts used in this study and the discovery EWAS. Since the EWAS was performed in monozygotic twins genetic polymorphism in depression risk genes did not impact the investigated differences in depression symptomatology scores [17]. However, in the current follow-up study, performed in non-related individuals, complex genetic variation associated with depression scores may complicate replication of previous results. However, no strong mQTL interactions were identified for CpG2 (cg05777061) across human life course in blood [64]. The only identified mQTL on autosomes for CpG2 was trans-acting SNP rs2844278 (estimate =  − 0.25, *p* value = 4.54E−08) mapping to *OXR1* gene [64]. The mQTL interaction was reported to be significant only for middle-aged individuals [64]. Interestingly, *OXR1* has been reported to play an important role in neuronal maintenance, mitochondrial morphology, and regulation of aging processes [65]. For CpG1, mQTL data are not available as the site is not targeted by the 450K methylation array.

Apart from differences in cohort design (monozygotic twins vs population-based), this follow-up study carries other differences in comparison with the EWAS reporting the association between DNAm in *KLK8* and depression symptomatology [17]. Demographics of participants from this study (PRISME cohort) was markedly different from the MADT and LSADT *KLK8* discovery cohorts. All three cohorts (PRISME, MADT, and LSADT) originated from the Danish population; however, individuals from the PRISME sample were on average over 20 years younger than MADT, over 30 years younger than LSADT, and had significantly lower frequency of males. Adjusting for sex and age in the regression models should minimize impact of these demographic cohort differences on detecting the association between depression symptomatology and *KLK8* DNAm levels. However, if there are different effects across ages or between the sexes influencing this association, adjusting for potential confounding variables in each study separately may not be sufficient to reduce differences in these effects between cohorts. Another difference between this study and the EWAS is that in our analyses it was not possible to adjust the regression models for cellular heterogeneity. This limitation is due to use of pyrosequencing to measure DNAm levels at the two candidate CpG sites of *KLK8*, while genome-wide methylomic data are needed to estimate blood cell proportions [57]. Moreover, different instruments were used to measure depression symptomatology across studies, ‘SCAN section 6-Depressed mood and ideation’ for PRISME vs affective depression assessment adapted from the ‘Depression Section of the Cambridge Mental Disorders of the Elderly Examination’ (CAMDEX) for MADT and LSADT. Additionally, environmental exposures, such as socioeconomic factors, lifestyle factors (tobacco smoking, diet, level of physical exercise), or medication use, are reported to influence both DNAm levels across the genome and depression symptomatology [16, 66]. Since in this study information on environmental exposures for investigated individuals was not available, we were not able to determine to what extend these additional factors explain variability in DNAm levels and studied depression-related phenotypes.

Despite the differences in cohort characteristics and the information available, results from both studies point to an association between epigenetic variation in the *KLK8* promoter region and severity of depressive symptoms. This was supported by additional analyses of our findings in four independent methylomic cohorts, where DNAm variation at 7 CpG sites located in the *KLK8* promoter region was associated with depression symptomatology in the GSMS cohort, but not in the other cohorts. This finding is of high interest as GSMS is the cohort that closest resembles the EWAS twin cohort [17]—its methylomic data originate from blood samples and the measured trait is severity of depression symptomatology. These results support previous findings on depression, where DNAm variation in the *KLK8* promoter and its expression levels were associated with severity of depression (recurrent vs first episode), but not with major depressive disorder diagnosis [17–19, 62]. Further, exploration of these findings by combining epigenomic, genomic, transcriptomic, and metabolomic data form large cohorts to study depression symptomatology will provide deeper understanding of the role of *KLK8* in the molecular pathophysiology of depression phenotypes.

## Conclusions

Our results suggest that blood-derived epigenetic variation of *KLK8* is associated with severity of depressed mood among depression cases, but does not allow for stratification of depression cases from controls. These results highlight the importance of investigation the intermediate phenotypes in complex trait, including symptomatology of depression.

## Supplementary Information

**Additional file 1:** **Figure S1.** Overview of pyrosequencing primer design to quantify *KLK8* DNAm levels at CpG1 and CpG2.

**Additional file 2:** **Supplementary Table 1.** Conditions for PCR amplification of *KLK8* promoter region containing CpG1 and CpG2 for pyrosequencing analysis. **Supplementary Table 2.** Overview of sex and age distribution of PRISME individuals after quality control filtering for each tested CpG site. **Supplementary Table 3.** Power calculations for PRISME, NESDA, GSMS, BioMom, and BrainMDD cohorts to replicate the association between *KLK8* DNAm levels and depression-related phenotypes.

**Additional file 3:** **Figure S2.** Overview of pyrosequencing primer location, genomic context of analyzed sites CpG1 and CpG2 from *KLK8* promoter region, and regulatory elements (CpG island and H3K27A mark).

**Additional file 4:** **Figure S3.** Overview of quality control processing of pyrosequencing data for *KLK8* DNAm levels at CpG1 and CpG2.

## Data Availability

According to Danish legislation, transfer and sharing of individual-level data requires prior approval from the Danish Data Protection Agency and requires that data sharing requests be dealt with on a case-by-case basis. For this reason, the data cannot be deposited in a public database. However, we welcome any enquiries regarding collaboration and individual requests for data sharing.
