# Supplementary material for: DNA methylation of the KLK8 gene in depression symptomatology
Source: Clin Epigenetics. 2021 Oct 29;13:200. doi: 10.1186/s13148-021-01184-5 (PMC8556955; doi:10.1186/s13148-021-01184-5)
Supplement: Supplementary file 1 — Additional file 1: Figure S1. Overview of pyrosequencing primer design to quantify KLK8 DNAm levels at CpG1 and CpG2. [file 13148_2021_1184_MOESM1_ESM.pdf]

5´ AACACTGGGTGTGAGTGAGAAGGGGCGGAGGGGATTGAACGTGAAATCTTGAGGGA 3´

Pyrosequencing primer
CpG sites

**Sequence to analyse before bisulfite conversion**	5´ GGGCGGAGGGGATTGAACGT 3´
**Sequence to analyse before bisulfite conversion including the bisulfite control C**	5´ GGGCGGAGGGGACTTGAACGT 3´
**PCR Forward Primer**	5´ GAT TTT GGA GTT TTT TAA TTG GGA A 3´
**PCR Reverse Primer**	5´ [BIO] ATC CCT CCT CTC CCT AAC CTC 3´
**Sequencing Primer**	5´ GTG AGT GAG AAG 3´
