# Supplementary material for: DNA methylation of the KLK8 gene in depression symptomatology
Source: Clin Epigenetics. 2021 Oct 29;13:200. doi: 10.1186/s13148-021-01184-5 (PMC8556955; doi:10.1186/s13148-021-01184-5)
Supplement: Supplementary file 2 — Additional file 2: Supplementary Table 1. Conditions for PCR amplification of KLK8 promoter region containing CpG1 and CpG2 for pyrosequencing analysis. Supplementary Table 2. Overview of sex and age distribution of PRISME individuals after quality control filtering for each tested CpG site. Supplementary Table 3. Power calculations for PRISME, NESDA, GSMS, BioMom, and BrainMDD cohorts to replicate the association between KLK8 DNAm levels and depression-related phenotypes. [file 13148_2021_1184_MOESM2_ESM.docx]

Supplementary Table 1. Conditions for PCR amplification of neuropsin promoter region containing CpG1 and CpG2 for further pyrosequencing.

Initial activation step: 15 min 95°C

3-step cycling

Denaturation: 30 sec 94°C

Annealing: 30 sec 56°C

Extension: 30 sec 72°C

Number of cycles: 36

Final extension: 10 min 72°C

Supplementary Table 2. Overview of sex and age distribution of PRISME individuals after quality control filtering for each tested CpG site.

|  | | |  |
| --- | --- | --- | --- |
|  | **Cases** | **Controls** | **P-value** |
| **Sex (%males) for CpG1** | 19% | 18% | 1^a^ |
| **Sex (%males) for CpG2** | 17% | 21% | 0.7^a^ |
| **Mean age in years (sd) for CpG1** | 46.2 (9.3) | 42.4 (9.4) | 0.06^b^ |
| **Mean age in years (sd) for CpG2** | 45.9 (9.4) | 43.6 (10.1) | 0.17 ^b^ |

^a^ Chi-square test; ^b^ generalized linear regression

Supplementary Table 3. Power calculations for PRISME, NESDA, GSMS, BioMom, and BrainMDD cohorts to identify the association between *KLK8* DNAm levels and depression-related phenotypes.

|  | Sample size | Alpha | Proportion of variance (r2) | Power |
| --- | --- | --- | --- | --- |
| PRISME_CpG1 | 87 | 0.05 | 0.01 | 0.15 |
| PRISME_CpG1 | 87 | 0.05 | 0.02 | 0.256 |
| PRISME_CpG1 | 87 | 0.05 | 0.03 | 0.361 |
| PRISME_CpG1 | 87 | 0.05 | 0.04 | 0.461 |
| PRISME_CpG1 | 87 | 0.05 | 0.05 | 0.552 |
| PRISME_CpG2 | 133 | 0.05 | 0.01 | 0.208 |
| PRISME_CpG2 | 133 | 0.05 | 0.02 | 0.368 |
| PRISME_CpG2 | 133 | 0.05 | 0.03 | 0.515 |
| PRISME_CpG2 | 133 | 0.05 | 0.04 | 0.64 |
| PRISME_CpG2 | 133 | 0.05 | 0.05 | 0.741 |
| NESDA | 1132 | 0.05 | 0.01 | 0.922 |
| NESDA | 1132 | 0.05 | 0.02 | 0.998 |
| NESDA | 1132 | 0.05 | 0.03 | 1 |
| NESDA | 1132 | 0.05 | 0.04 | 1 |
| NESDA | 1132 | 0.05 | 0.05 | 1 |
| GSMS | 1034 | 0.05 | 0.01 | 0.897 |
| GSMS | 1034 | 0.05 | 0.02 | 0.996 |
| GSMS | 1034 | 0.05 | 0.03 | 1 |
| GSMS | 1034 | 0.05 | 0.04 | 1 |
| GSMS | 1034 | 0.05 | 0.05 | 1 |
| BioMom | 1400 | 0.05 | 0.01 | 0.964 |
| BioMom | 1400 | 0.05 | 0.02 | 1 |
| BioMom | 1400 | 0.05 | 0.03 | 1 |
| BioMom | 1400 | 0.05 | 0.04 | 1 |
| BioMom | 1400 | 0.05 | 0.05 | 1 |
| BrainMDD | 206 | 0.05 | 0.01 | 0.298 |
| BrainMDD | 206 | 0.05 | 0.02 | 0.528 |
| BrainMDD | 206 | 0.05 | 0.03 | 0.705 |
| BrainMDD | 206 | 0.05 | 0.04 | 0.827 |
| BrainMDD | 206 | 0.05 | 0.05 | 0.903 |
