# Supplementary material for: DNA methylation of the KLK8 gene in depression symptomatology
Source: Clin Epigenetics. 2021 Oct 29;13:200. doi: 10.1186/s13148-021-01184-5 (PMC8556955; doi:10.1186/s13148-021-01184-5)
Supplement: Supplementary file 3 — Additional file 3: Figure S2. Overview of pyrosequencing primer location, genomic context of analyzed sites CpG1 and CpG2 from KLK8 promoter region, and regulatory elements (CpG island and H3K27A mark). [file 13148_2021_1184_MOESM3_ESM.pdf]

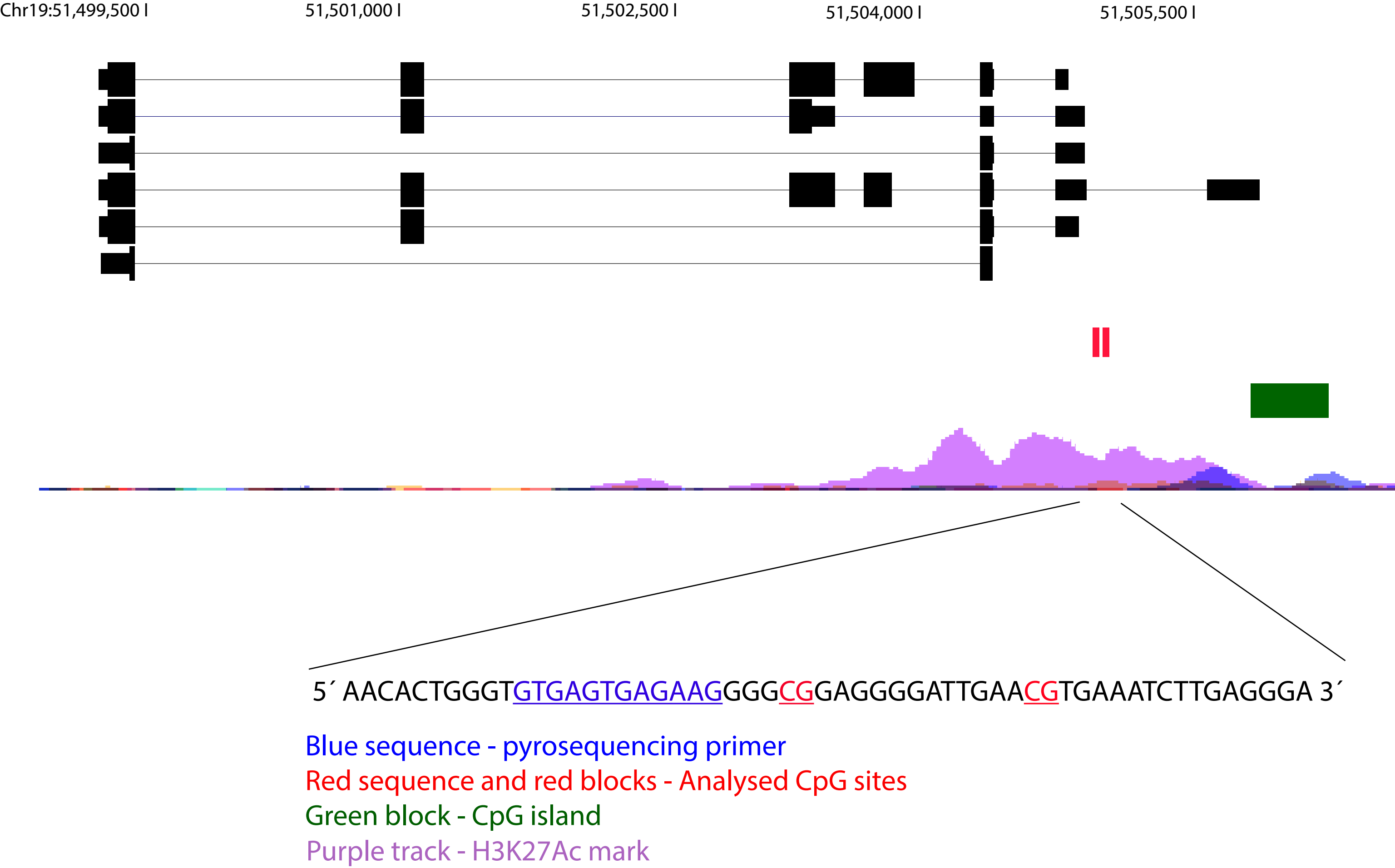
Chr19:51,499,500 I
51,501,000 I
51,502,500 I
51,504,000 I
51,505,500 I
5´ AACACTGGGTGTGAGTGAGAAGGGGCGGAGGGGATTGAACGTGAAATCTTGAGGGA 3´
Blue sequence - pyrosequencing primer
Red sequence and red blocks - Analysed CpG sites
Green block - CpG island
Purple track - H3K27Ac mark
