# Supplementary figures and images for: DNA methylation of the KLK8 gene in depression symptomatology
Source: Clin Epigenetics. 2021 Oct 29;13:200. doi: 10.1186/s13148-021-01184-5 (PMC8556955; doi:10.1186/s13148-021-01184-5)

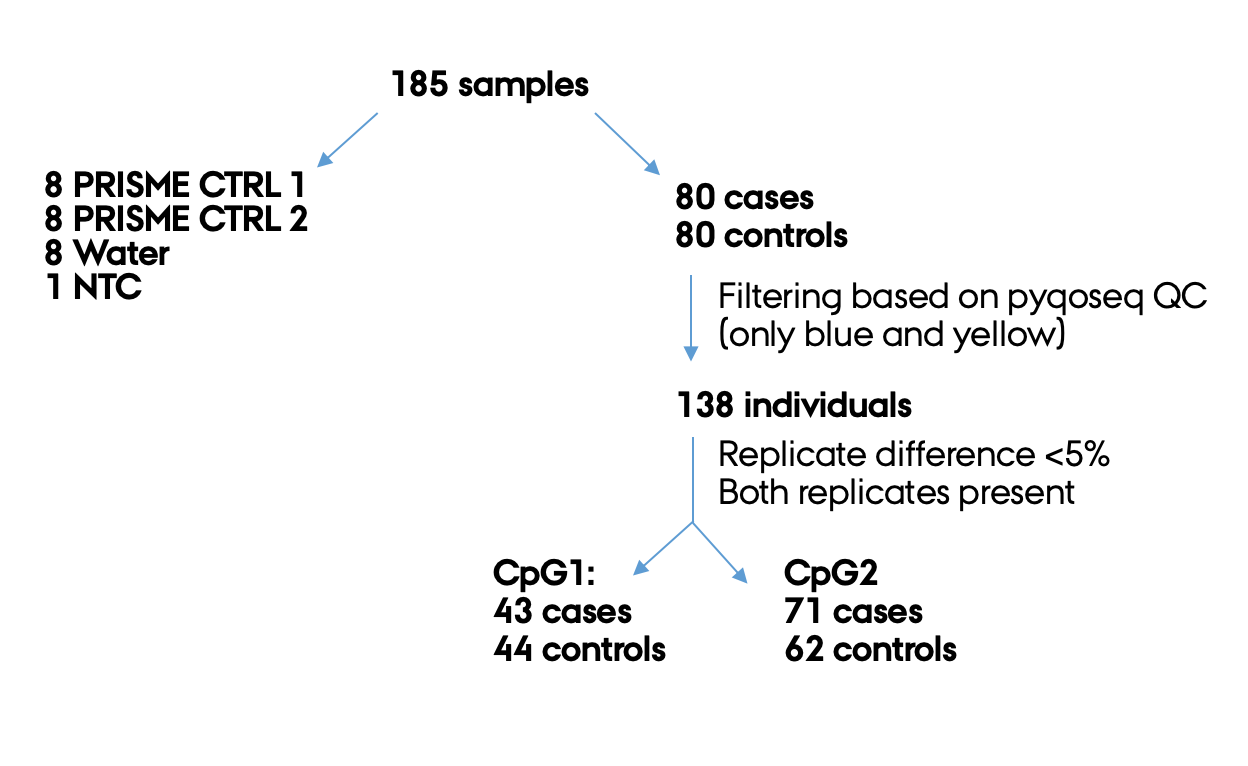

Supplement: Supplementary file 4 — Additional file 4: Figure S3. Overview of quality control processing of pyrosequencing data for KLK8 DNAm levels at CpG1 and CpG2. [file 13148_2021_1184_MOESM4_ESM.png]
